# Extradural spinal neurocysticercosis mimicking epidural abscess: a case report

**DOI:** 10.1097/MS9.0000000000005119

**Published:** 2026-05-06

**Authors:** Sameer Lamichhane, Dinesh Kafle, Sushil Poudel, Sabik Raj Kayastha, Sneha Dabadi, Sujal Rajbhandari, Anupa Khadka

**Affiliations:** a Department of Orthopedic and Trauma Surgery, Tribhuvan University Teaching Hospital, Kathmandu, Nepal; b Maharajgunj Medical Campus, Tribhuvan University, Institute of Medicine, Kathmandu, Nepal; c Department of Dermatology and Venereology, Patan Academy of Health Science, Lalitpur, Nepal

**Keywords:** case report, extradural, mimicking, multidisciplinary, neurocysticercosis, rare

## Abstract

**Introduction:**

Neurocysticercosis is an infection of the central nervous system (CNS) caused by the larvae of the tapeworm *Taenia solium*. Spinal neurocysticercosis is a very uncommon form of neurocysticercosis, among which intradural extramedullary is the most common, intramedullary is less common, and extradural is very rare.

**Case presentation:**

A 28-year-old woman presented to the center with complaints of back pain for 2 years; gradual-onset bilateral lower limb weakness associated with a loss of sensation and stiffness of the lower limbs for 2 months; intermittent fever with a maximum recorded temperature of 101 °F; and involuntary passage of urine and stool for 3 days, with no history of trauma or any other comorbidities.

**Discussion:**

Extradural spinal neurocysticercosis is a very rare form of neurocysticercosis, presenting with very vague symptoms that may mimic other infectious conditions of the spinal cord. The patient was diagnosed with an epidural abscess, underwent decompression surgery, and was treated accordingly in the initial phase, as MRI findings suggested infective pathology. However, the histological diagnosis of the tissue after surgery was suggestive of neurocysticercosis, and medical therapy for the same was then introduced, with the patient continuing rehabilitation. This multidisciplinary approach to treatment is important for the proper management of the patient.

**Conclusion:**

In summary, this case highlights a rare presentation of extradural spinal neurocysticercosis. It sheds light on the diagnostic challenges and treatment, emphasizing the importance of considering neurocysticercosis in patients with spinal symptoms mimicking infective pathology.

## Introduction

Neurocysticercosis is an infection of the central nervous system caused by the larvae of *Taenia solium*. It is the most common parasitic infection of the CNS^[^1^]^. According to the WHO, it is an endemic disease in South America, Eastern Europe, Southeast Asia, Sub-Saharan Africa, and the Indian subcontinent, with moderate prevalence in Nepal^[^2^]^. It is transmitted through the ingestion of eggs of *T. solium* via the fecal–oral route. The larvae, or cysticerci, may infect the brain parenchyma, subarachnoid space, ventricular system, or spinal cord. Almost 70% of patients with neurocysticercosis present with seizures as the primary or even the sole manifestation^[^3^]^.

HIGHLIGHTS

- Extradural spinal neurocysticercosis is an extremely rare manifestation of central nervous system infection.
- Clinical and MRI findings may closely mimic a spinal epidural abscess.
- Histopathology remains crucial for a definitive diagnosis in atypical spinal lesions.
- Combined surgical decompression and medical therapy improve neurological outcomes.
- Neurocysticercosis should be considered in endemic regions with spinal cord compression.

Spinal neurocysticercosis is a very uncommon form of the disease, reported in only 1–6% of all cases diagnosed with neurocysticercosis^[^4^]^. In spinal neurocysticercosis, the cysticerci typically infect the intradural intramedullary or intradural extramedullary regions^[^5^]^. Among the cases of spinal cysticercosis, the extradural type is the least common presentation. There are very few reported cases of extradural neurocysticercosis^[^6^]^.

In our case, the patient presented with symptoms similar to a spinal epidural abscess, which is the collection of purulent material in the epidural space due to an infection. Spinal epidural abscess is caused by bacteria and is quite rare in itself, with only 0.2–2 cases per 10 000 hospital admissions^[^7^]^. Thus, extradural neurocysticercosis appearing as a spinal epidural abscess makes this an intriguing case. MRI is considered the gold standard for diagnosis, along with histopathological confirmation^[^8^]^. Treatment mostly includes medical management, but surgical intervention is indicated if severe neurological deficits are present^[^5^]^.

In this study, we present a case of an adult woman initially treated for a spinal epidural abscess, who underwent decompression surgery and was later diagnosed with neurocysticercosis from a histopathological exam. This case has been reported in line with the SCARE 2025 guidelines^[^9^]^.

## Clinical presentation

The patient, a 28-year-old woman, presented to our center with complaints of back pain, a gradual onset of bilateral lower limb weakness for 2 months, intermittent fever reaching a maximum of 101 °F, and involuntary passage of urine and stool for 3 days. The weakness was associated with a loss of sensation and stiffness in the lower limbs. There was no history of trauma, comorbidities, significant medical conditions, surgery, drug use, or allergy. Family history was insignificant.

On examination, the patient was conscious, alert, and oriented to time, place, and person. There were pressure sores over the sacrum and bilateral lower limbs. Upon neurological examination, the tone of both lower limbs was increased. The power was Grade 1 according to the Medical Research Council grading for each limb. Hyperreflexia was present in both the knee and ankle joints with sustained clonus. The plantar reflex was diminished on both sides. Sensory examination revealed decreased sensation below the level of T10 and no sensation below the level of L4. On per rectal examination, the anal tone was lax, voluntary anal contraction was absent, and perianal sensation and deep anal stimulus were absent. There was no spinal tenderness. The motor and sensory functions of the bilateral upper limbs were preserved.

In the CBC investigation, the leukocyte count was 8400; there was an increased neutrophil count (76%) and decreased lymphocyte count (17%), and the patient had decreased hemoglobin (10.3 g/dL) and red cell count (3.4 million/µL). The C-reactive protein was highly elevated (59.21 mg/L) [Range: 0–6]. The patient was admitted for further workup.

**Figure 1.**
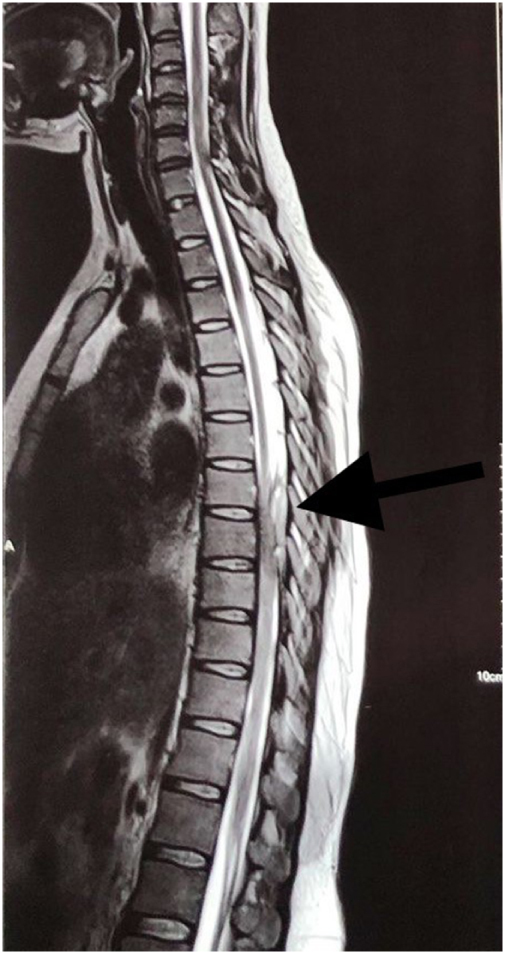
Sagittal T2-weighted MRI of the spine shows a posterior extradural lesion at the T7–T9 level, producing severe spinal canal stenosis with anterior displacement of the spinal cord. Source: Author’s own image. Published with written informed consent from the patient.

**Figure 2.**
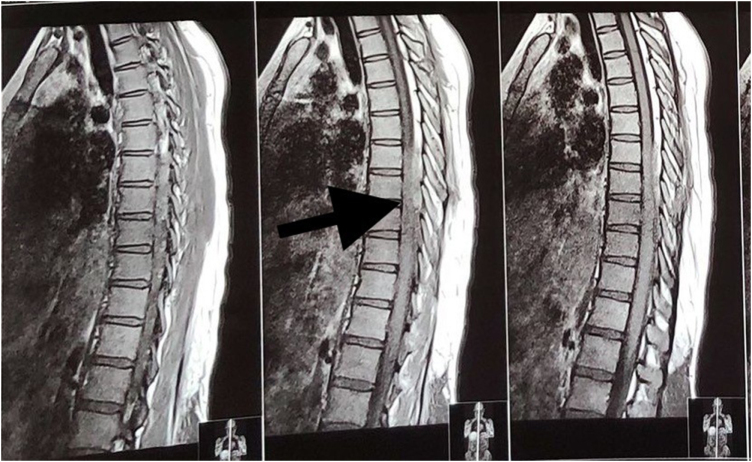
Sagittal T1-weighted MRI highlighting the extradural lesion compressing and deviating the spinal cord at T7–T9. Source: Author’s own image. Published with written informed consent from the patient.

Subsequently, an MRI of the dorsal spine with screening of the whole spine was performed. It revealed a posterior epidural lesion at the T7–T9 vertebral level, causing severe stenosis of the central spinal canal with compression and leftward displacement of the spinal cord (Figs 1 and 2). Patchy areas of marrow edema along the posterior aspect of the T8–T11 vertebrae were noticed. The features were suggestive of an infective pathology. A diagnosis of an epidural abscess was made, and the patient was planned for surgery.

**Figure 3.**
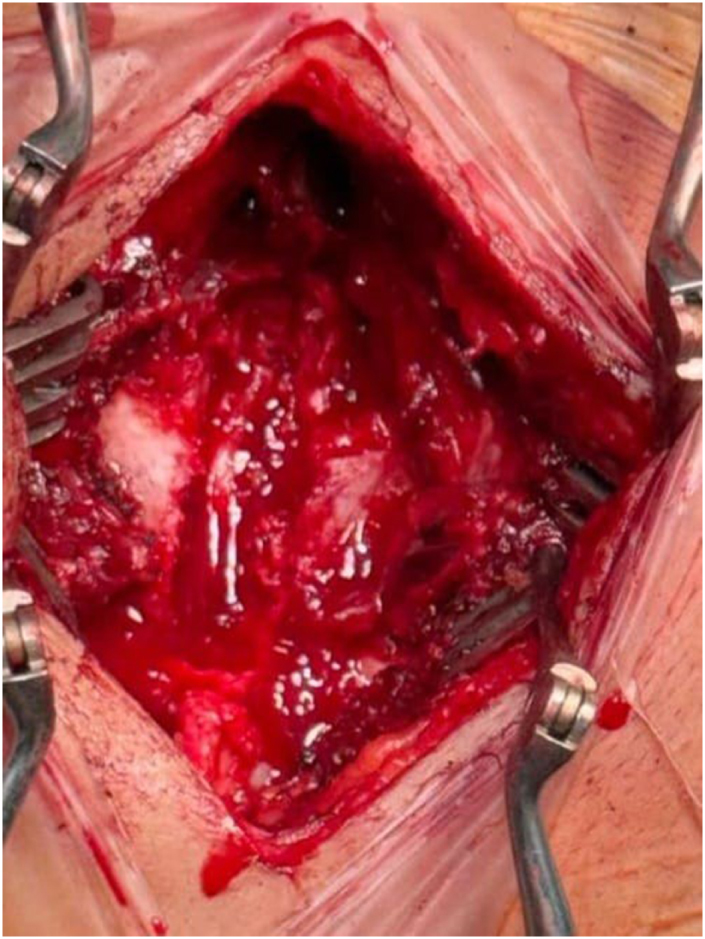
Intraoperative picture after central decompression, showing a thickened membrane over the spinal cord. Source: Author’s own image. Published with written informed consent from the patient.

**Figure 4.**
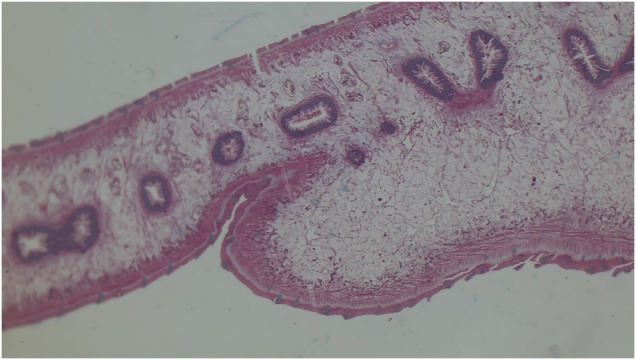
Histopathological section showing cysticercosis with a parasitic cyst wall, dense mixed inflammatory infiltrate composed of lymphocytes, plasma cells, neutrophils, and eosinophils, and proliferating blood vessels in fibrocollagenous tissue (H&E stain). Source: Author’s own image. Published with written informed consent from the patient.

**Figure 5.**
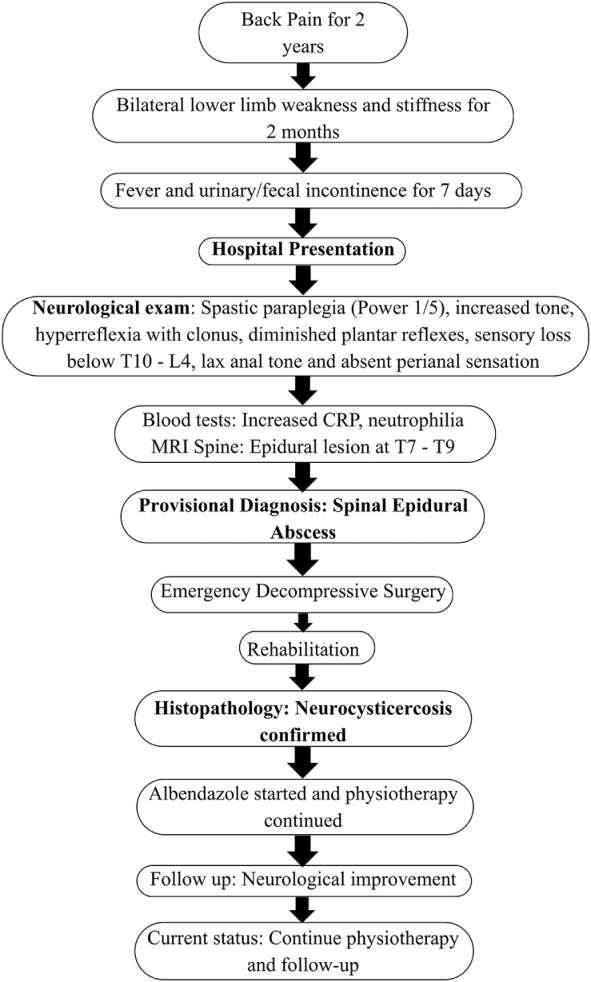
Flowchart of symptom onset to diagnosis and treatment in the present case. Source: Created by the authors based on the patient’s clinical course.

The patient underwent an emergency decompression surgery. The pathology was identified, and an organized collection above the spinal cord was meticulously scraped (Fig. 3). The collection was sent for culture, GeneXpert, and biopsy. After the surgery, the patient condition was managed with antibiotics and analgesics. The culture, as well as the GeneXpert result, came back negative. The patient was later discharged and referred to the Spinal Injury Rehabilitation Center for rehabilitation and was advised to follow up with the biopsy reports. Later, the biopsy report confirmed the diagnosis to be neurocysticercosis (Fig. 4). Subsequently, a definitive treatment with albendazole was administered. On follow-up, the patient was improving but had not developed sensations in the legs yet, and movement was also restricted. The patient could walk only with support and had regained control over urine retention; however, fecal incontinence was still present. There were no complaints regarding fever or any new symptom. The surgical wound had healed, and the patient was continuing her course of physiotherapy. The patient’s clinical course from symptom onset to diagnosis and treatment is summarized in Figure 5.

## Discussion

Neurocysticercosis, the most common parasitic infection of the CNS, is caused by the larvae of a tapeworm, *T. solium*. This tapeworm is prevalent in areas with poor sanitation and hygiene. When a person consumes food or water contaminated with eggs, they reach the gastrointestinal tract. From there, they evolve into oncospheres that are carried through the bloodstream into the CNS and other tissues, where they mature into larval forms^[^1^]^. Neurocysticercosis is most commonly seen within the cranium, making spinal neurocysticercosis a rare presentation of the condition^[^10^]^. In spinal neurocysticercosis, the hematogenous spread of larvae through the cerebrospinal pathway leads to the formation of a cyst within the spinal cord and its surrounding spaces^[^11^]^. Intradural extramedullary involvement is the most frequent site of infestation, accounting for 76.92% of cases, often involving the thoracic, cervical, or lumbosacral regions, while intramedullary involvement is less common, and extradural involvement is even rarer^[^12–14^]^. We could identify only three instances of extradural involvement of cysticercosis in the existing literature (Table 1)^[^6,15,16^]^.

**Table 1:** Comparison of past cases of extradural spinal neurocysticercosis.

Case	Age/Sex	Clinical features	Diagnosis	Outcome
PMID: 201 878	25Y/M	Low backache with radiculopathy	Myelogram	Improved
Mohanty *et* *al*^[^6^]^	55Y/M	Spastic tetraparesis	Myelogram, CT scan, Histopathology	Improved
PMID: 21 423 911	19Y/M	Headache, vomiting, seizures, decreased vision, proptosis (disseminated neurocysticercosis)	MRI, biopsy	-
Present case	28Y/F	Lower back pain, paraplegia, urinary and bowel incontinence	MRI of spine and histopathology	Improved

Clinical manifestations depend on the location of the lesion and its mass effect. Symptoms are very vague, including motor deficits, sensory disturbances, and pain localized to the neck, back, or limbs, which mimic spinal cord tumors and other causes of intradural or intramedullary cystic lesions^[^10,12,14,17^]^. In this case, bilateral lower limb weakness associated with a loss of sensation and stiffness of the lower limbs, fever, and fecal and urinary incontinence was observed, which misled the diagnosis at the initial stage.

Diagnosis of neurocysticercosis is usually performed through neuroimaging and confirmed by serology, as histological confirmation is not always feasible in all cases. The morphology and localization of cysts, the burden of infection, the stage of the cysts, and the presence of surrounding inflammation can be observed in CT and MRI scans. The enzyme-linked immunoelectrotransfer blot (EITB) assay, which uses lentil lectin–purified glycoprotein antigens (LLGP) to detect antibodies to *T. solium* in serum, is documented as the most reliable serology test^[^18^]^. As similar clinical findings and radiological patterns can be observed in other conditions like tuberculosis, tumors, abscesses, or other infections, radiological diagnosis alone may lead to misinterpretation. Therefore, biopsy and histopathological examination should also be considered in endemic regions, even if the symptoms strongly suggest an infective pattern.

In many cases of spinal neurocysticercosis, it is not the first diagnosis made or even suspected. In multiple cases, other diagnoses were made and later turned out to be neurocysticercosis after serology or histopathology^[^5^]^.

The treatment includes medical therapy with albendazole (15 mg/kg/day), often in combination with corticosteroids, with the duration determined according to the clinical response and imaging findings. Symptoms of patients and MRI images should be monitored closely for regression of cystic lesions and improvement in enhancement^[^19^]^. Surgical intervention is considered for patients who have progressive or severe neurological deficits, medical therapy failure, and uncertainty in diagnosis. Surgery may also be required for decompression in cases of cord compression, as in this case. However, a combined treatment with surgery followed by cysticidal and corticosteroid administration seems to be superior to surgery or medical treatment in isolation and appears to provide the highest chances of recovery^[^12^]^. Our patient had cord compression caused by the lesion; hence, surgical therapy was chosen for treatment.

This case presents the rare manifestation of extradural spinal neurocysticercosis, emphasizing the importance of considering parasitic infections like neurocysticercosis in endemic regions for spinal cord pathology, even if symptoms, clinical signs, and radiological findings suggest infective pathology.

## Conclusion

Although neurocysticercosis is the most common parasitic infection of the CNS, its spinal involvement is less common, and extradural manifestation is very rare. The non-specific presentation of spinal neurocysticercosis makes it difficult to diagnose, so different diagnostic modalities should be considered, including radiological and histopathological methods. Surgical management, along with medical therapy, is considered effective for treatment. Multidisciplinary management and timely rehabilitation can improve neurological symptoms.

## Ethical approval

Ethical approval was not required for this study as per institutional guidelines.

## Data Availability

All data supporting the findings of this study are included within the article. Additional data are available from the corresponding author upon reasonable request.

## Notes

The authors declare that this study has no funding source.

## Statement on previous or redundant publication

The authors declare that there are no previous reports, submissions, or publications that overlap with the content of this manuscript.
